# Cephalometric Study of Occipital Spurs and Their Association With Various Malocclusions and Facial Patterns

**DOI:** 10.7759/cureus.113103

**Published:** 2026-07-21

**Authors:** Uday H Barhate, Kavita Negi, Ritu Duggal, Balachandran Rajiv, Prabhat K Chaudhari, Vilas Samrit

**Affiliations:** 1 Orthodontics and Dentofacial Deformities, All India Institute of Medical Sciences, New Delhi, New Delhi, IND; 2 Centre for Dental Education and Research, All India Institute of Medical Sciences, New Delhi, New Delhi, IND; 3 Orthodontics, All India Institute of Medical Sciences, New Delhi, New Delhi, IND

**Keywords:** lateral cephalogram, occipital bone, skull morphology, spur length, spur types

## Abstract

Purpose: Occipital spurs (OS), bony outgrowths from the external occipital protuberance, are increasingly observed in young adults and have been associated with headache and neck pain. However, the available literature on this condition remains limited. This study aimed to determine the prevalence, morphological variations, and lengths of OS and assess their associations with gender, skeletal malocclusion, vertical facial patterns, overjet, and overbite in an Indian population using lateral cephalograms.

Methodology: A retrospective cross-sectional study was conducted on 180 lateral cephalometric radiographs of individuals aged 18-24 years. OS were identified and classified as type 1 (flat), type 2 (crest), and type 3 (spine). Spur length was measured from its highest point to the most distant point from the cranial contour. Radiographs were grouped by gender, skeletal malocclusion (class I-III), vertical growth patterns, overjet, and overbite. Statistical analyses were performed using SPSS v26 (IBM Corp., Armonk, NY).

Results: OS were present in 65% of cases (117/180), with type 2 being most frequent (41.6%), followed by type 1 (17.2%) and type 3 (6.1%). Males showed slightly higher prevalence (35.55%) than females (29.44%) without statistical significance (p = 0.16). No significant associations emerged between OS type and sagittal skeletal malocclusion (p = 0.08), facial patterns (p = 0.37), or overjet (p = 0.07). However, spur length was significantly greater in deep bite individuals (10.24 ± 2.37 mm) than in those with normal overbite (8.64 ± 2.19 mm; p = 0.007).

Conclusion: Type 2 OS was the most prevalent morphology across groups. Increased spur length in deep bite cases suggests a possible link with vertical skeletal relationships or postural factors, warranting further prospective studies.

## Introduction

The cranial base articulates with the maxillary and mandibular jaw and can directly influence facial morphology. Numerous studies have been conducted to investigate the relationship between cranial morphology and facial structures [1-4]. Craniofacial features vary across different types of malocclusions. In class I malocclusion, the cranial base length and angle are within the normal limits, and the maxilla and mandible are in a relatively harmonious anteroposterior relationship, though dental malalignment may be present. Class II often features a reduced cranial base angle and increased anterior length, contributing to mandibular retrusion or maxillary protrusion and a convex profile [4]. On the other hand, class III malocclusions often have a shorter and more acute anterior cranial base, which may contribute to a more prognathic mandible or a retrusive maxilla, leading to a concave facial profile [5,6]. Variations in cranial base morphology thus not only correlate with sagittal jaw discrepancies but may also influence the vertical facial dimensions and the overall aesthetic balance of the face [7]. Understanding these relationships is critical for orthodontic diagnosis and treatment planning, particularly in growing patients where skeletal modifications are more feasible.

Cephalometric radiographs are routinely utilized in orthodontic treatment, facilitating the assessment of craniofacial and dental structures, growth, and morphometric relationships. While most orthodontic patients are typically young and healthy, these radiographs may unexpectedly reveal pathological conditions or developmental abnormalities, as well as anatomical variations resembling pathological conditions [8].

The external occipital protuberance (EOP), found on the posterior surface of the occipital bone, serves as the attachment site for the ligamentum nuchae and trapezius muscle, with its highest point known as the inion [9]. In recent years, there has been an increased detection of enlarged EOP in radiographic examinations of young individuals [10-12]. Occipital spurs (OS), considered anatomical variations, are characterized by abnormal enlargement of the posterior occipital protuberance. Broca initially categorized EOP into six anatomical types, later streamlined by Gülekon and Turgut into three subtypes: smooth, crest form, and spine-like configuration [13,14].

Several skeletal dysplasias and syndromes have been associated with projections of the cranium's occipital region, highlighting the importance of understanding variations in this area [15]. While typically considered a normal variation, OS can manifest symptoms that are concerning for patients, including tender bony swelling at the back of the neck, discomfort, pain at rest and during neck movements extending to the shoulder area, and occasional recurrent occipital headaches [16,17]. These symptoms often arise during late adolescence, coinciding with growth spurts, as the protuberance enlarges, inducing subperiosteal stretching and tenderness. Surgical removal of the hyperostotic area and smoothing of the bone are effective in alleviating symptoms and improving aesthetic appearance in individuals experiencing discomfort due to enlarged OS [16-18].

Nevertheless, there is a paucity of current literature examining the occurrence of OS in relation to craniofacial morphology. Cheng et al. analyzed the correlation between spur length, gender, and age, as well as explored the relationship between occipital spur length and craniofacial structure [19]. Generally, males exhibit longer spur lengths than females, and individuals under 18 years of age have shorter spur lengths than adults. Moreover, subjects with enlarged OS tend to show greater linear craniofacial measurements compared to those with regular OS [19]. Most existing studies, however, have focused on Turkish and Chinese populations. Data from the Indian population remain scarce despite potential differences in craniofacial characteristics across ethnic groups. Consequently, the principal aim of this study is to comprehensively explore the association between OS and various craniofacial structures in the Indian population, thereby addressing a critical gap in the current literature and providing population-specific insights that may improve diagnostic and anthropometric understanding.

## Materials and methods

The study was approved by the Institute Ethics Committee, All India Institute of Medical Sciences, New Delhi (Approval No. AIIMSA1493/09.07.2024). The sample size was determined using the expected proportion between the variables, with values estimated from the literature [19]. Assuming 80% power, a type I error of 5%, and a degree of association of 0.22 between the variables, a minimum sample size of 60 subjects per group was calculated to provide adequate external validity for the present study.

Lateral cephalometric radiographs were obtained from patients reporting to the department of orthodontics and dentofacial deformities. All radiographs were acquired using a NewTom cephalometric unit (NewTom GiANO HR, Imola, Italy) with standardized exposure parameters of 66 kVp, 10 mA, and 10.5 s. Only high-quality cephalograms with well-defined radiographic landmarks were included. Radiographs of individuals aged 18-24 years, in which the entire occipital bone was visible, were selected. Cephalograms with positioning errors, clefts or other craniofacial malformations, and the presence of cysts or tumors were excluded.

The cephalograms were categorized into skeletal class I, class II, and class III malocclusions based on the A point-Nasion-B point (ANB) angle (0-4°, >4°, and <0°, respectively) [20]. In addition, vertical facial growth patterns were assessed using the Frankfort-mandibular plane angle (FMA). According to Tweed's classification, the cephalograms were further categorized into hypodivergent (FMA <22°), normodivergent (FMA 22-28°), and hyperdivergent (FMA >28°) facial types [21].

Analysis of cephalometric landmarks

All cephalograms were uploaded and analyzed independently by two orthodontists using Dolphin Imaging 11.95 Premium software (Dolphin Imaging & Management Solutions, Chatsworth, CA). The definitions of the cephalometric landmarks and measurements are presented in Table 1.

**Table 1:** Angular and linear cephalometric measurements for craniofacial evaluation. SN plane: Sella-Nasion plane; MP: mandibular plane; FH: Frankfort horizontal.

Cephalometric parameter	Definition
Sella Nasion Point A (SNA)	Angle between the SN plane and the Nasion-A point line.
Sella Nasion Point B (SNB)	Angle between the SN plane and the Nasion-B point line.
Point A Nasion Point B (ANB)	Angle between the Nasion-A point line and the Nasion-B point line.
Frankfurt mandibular plane angle (MP-FH)	Angle formed by the mandibular plane and Frankfurt plane.
Overjet	Horizontal distance in millimeters, extending from the labial surface of the mandibular central incisor to the labial surface of the most prominent maxillary incisor.
Overbite	Vertical distance in millimeters, representing the total crown length of mandibular incisors that is overlapped by maxillary incisors.

**Figure 1:**
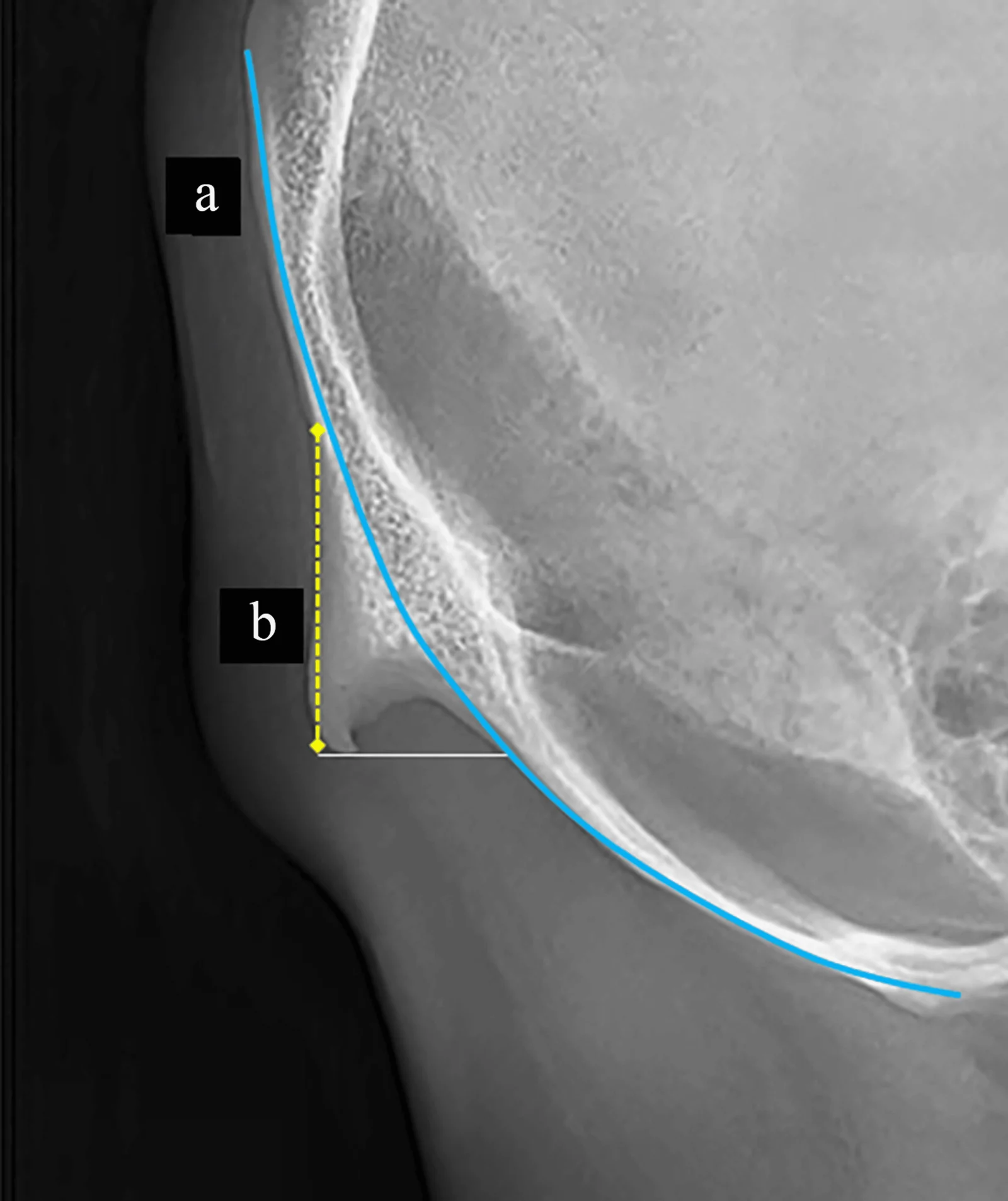
Measurement of occipital spur length. (a) Contour of skull. (b) Length of the spur.

A hyper-radiopaque outline delineating the cranium was identified, corresponding to its natural contour. Any projection extending more than 5 mm beyond this contour was considered an occipital spur [12]. The lateral cephalograms were subsequently categorized according to skeletal malocclusion and vertical facial growth pattern. The distance from the highest point of the occipital spur to its most prominent point was defined as the spur length (Figure 1).

**Figure 2:**
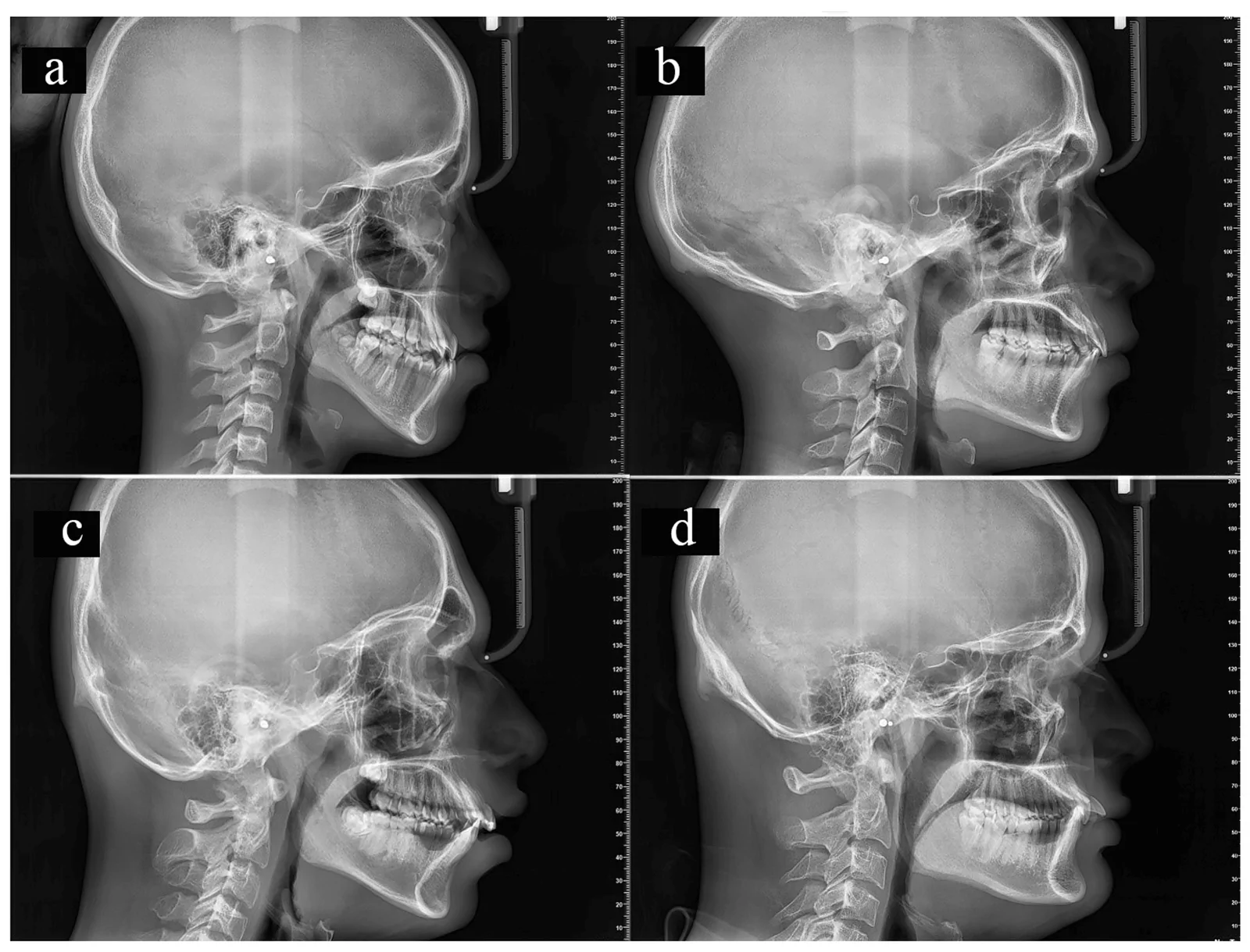
Types of occipital spurs. (a) Absence of occipital spur. (b) Type 1 (flat). (c) Type 2 (crest). (d) Type 3 (spine).

OS were further classified according to the classification proposed by Gülekon and Turgut as type 1 (flat), type 2 (crest), and type 3 (spine) (Figure 2) [13]. To assess intra-examiner reliability, all lateral cephalograms were reanalyzed after an interval of two weeks.

Statistical analysis

The collected data were compiled in a Microsoft Excel worksheet (Microsoft Corporation, Redmond, WA) and analyzed using SPSS software (IBM Corp., Armonk, NY). Descriptive statistics were used to summarize the data. Categorical variables were expressed as frequencies and percentages, whereas continuous variables were presented as mean ± standard deviation. The Shapiro-Wilk test was used to assess data normality. Frequencies and percentages between groups were compared using the chi-square test. Intergroup comparisons were performed using the Mann-Whitney U test, while comparisons among multiple groups were carried out using the Kruskal-Wallis test. Statistical significance was set at p < 0.05, with an alpha error of 5%, beta error of 20%, and study power of 80%.

## Results

A total of 180 lateral cephalograms were analyzed. The demographic and clinical characteristics of the included subjects are presented in Table 2.

**Table 2:** Distribution of cephalometric parameters among subjects. SNA: Sella-Nasion Point A; SNB: Sella-Nasion Point B; ANB: Point A Nasion Point B; FMA: Frankfort-mandibular plane angle; MP: mandibular plane; FH: Frankfort horizontal.

Cephalometric parameters	Observed values	
	Female (89)	Male (91)
SNA (°)	81.84 ± 4.59	82.39 ± 4.18
SNB (°)	80.14 ± 4.92	80.28 ± 5.34
ANB (°)	1.72 ± 4.86	2.11 ± 4.73
FMA (MP-FH) (°)	23.54 ± 6.95	22.02 ± 8.07
Overjet (mm)	3.10 ± 4.07	3.87 ± 4.46
Overbite (mm)	1.49 ± 2.91	2.06 ± 3.06
Age (years)	20.8 ± 1.80	21.09 ± 1.66

Analysis revealed the presence of OS in 117 cases, resulting in an overall prevalence of 65%. The distribution of spur types was as follows: type 1 (flat) in 31 cases, type 2 (crest) in 75 cases, and type 3 (spine) in 14 cases (Figure 2).

Males exhibited a slightly higher prevalence of OS (35.55%) than females (29.44%); however, this difference was not statistically significant (p = 0.162). Type 2 spurs were the most frequently observed subtype in both sexes, accounting for 64.1% of all OS cases (Table 3).

**Table 3:** Distribution of occipital spur types by gender, occlusion, and growth pattern. If the p-value is <0.05, then it is considered significant.

Parameters	Occipital spur				Total (%)	P-value
	Absent (%)	Type 1 (%)	Type 2 (%)	Type 3 (%)		
Gender						
Female	36 (20)	18 (10)	31 (17.22)	4 (2.22)	89 (49.4)	0.16
Male	27 (15)	13 (7.22)	44 (24.44)	7 (3.88)	91 (50.6)	
Total	63 (35)	31 (17.22)	75 (41.66)	11 (6.11)	180 (100)	
Occlusion						
Class I	19 (10.6)	4 (2.22)	34 (18.9)	3 (1.67)	60 (33.33)	0.08
Class II	20 (1.11)	14 (7.78)	23 (1.28)	3 (1.67)	60 (33.33)	
Class III	24 (1.33)	13 (7.22)	18 (10)	5 (2.78)	60 (33.33)	
Total	63 (35)	31 (17.2)	75 (41.7)	11 (6.11)	180 (100)	
Growth pattern						
Normodivergent	19 (10.6)	10 (5.55)	12 (6.66)	2 (1.11)	43 (23.88)	0.37
Hypodivergent	28 (15.55)	13 (7.22)	44 (24.44)	5 (2.78)	90 (50)	
Hyperdivergent	16 (8.88)	8 (4.44)	19 (10.55)	4 (2.22)	47 (26.11)	
Total	63 (35)	31 (17.2)	75 (41.7)	11 (6.11)	180 (100)	
Overjet						
Normal	25 (13.88)	12 (6.66)	44 (24.44)	6 (3.33)	87 (48.33)	0.07
Increased	25 (13.88)	9 (5)	23 (12.77)	2 (1.11)	59 (32.77)	
Reverse	13 (7.22)	10 (5.55)	8 (4.44)	3 (1.66)	34 (18.88)	
Total	63 (35)	31 (17.22)	75 (41.66)	11 (6.11)	180 (100)	
Overbite						
Normal	22 (12.22)	13 (7.22)	41 (22.77)	6 (3.33)	82 (45.55)	0.27
Deep bite	20 (11.11)	9 (5)	21 (11.66)	2 (1.11)	52 (28.88)	
Open bite	21 (11.66)	9 (5)	13 (7.221)	3 (1.66)	46 (25.55)	
Total	63 (35)	31 (17.22)	75 (41.66)	11 (6.11)	180 (100)	

The mean spur length was slightly greater in males (9.77 ± 2.45 mm) than in females (9.57 ± 2.54 mm), although this difference was not statistically significant (p = 0.47) (Table 4).

**Table 4:** Distribution of occipital spur length in various parameters. If the p-value is <0.05, then it is considered significant. KW: Kruskal-Wallis test; MWU: Mann-Whitney U test.

Variable	Group	Mean ± SD (mm)	KW p-value	Significant post-hoc (Dunn's test)
Gender	Male	9.77 ± 2.45	MWU = 0.47	-
	Female	9.57 ± 2.54		
Malocclusion	Class I	9.79 ± 2.05	0.65	
	Class II	9.50 ± 2.50		
	Class III	9.75 ± 2.97		
Vertical pattern	Hyperdivergent	9.15 ± 2.75	0.32	
	Hypodivergent	9.89 ± 2.47		
	Normodivergent	9.68 ± 2.31		
Overjet	Increased (>4 mm)	9.95 ± 2.46	0.38	
	Normal (0-4 mm)	9.14 ± 2.35		
	Reverse (<0 mm)	9.79 ± 2.72		
Overbite	Deep bite	10.24 ± 2.37	0.007	Deep bite vs. normal (p = 0.002, p bonf = 0.005)
	Normal	8.64 ± 2.19		-

With respect to skeletal malocclusion, class I subjects predominantly exhibited type 2 spurs (56.67%), whereas class II subjects demonstrated a more balanced distribution of spur types, with type 2 remaining the most common subtype (38.33%). Class III subjects showed the lowest prevalence of type 2 spurs (30%). Despite these variations in the distribution of spur types, no statistically significant difference in spur length was observed among the skeletal malocclusion groups (p = 0.65).

The distribution of occipital spur types across different vertical growth patterns was not significantly different (p = 0.37). Among hyperdivergent individuals, type 1 spurs were the most common (23.26%), followed by type 2 (27.91%) and type 3 (4.65%). In hypodivergent individuals, type 2 spurs were the most prevalent (48.89%), followed by type 1 (14.44%) and type 3 (5.56%). Normodivergent individuals also showed a predominance of type 2 spurs (40.43%), while type 3 spurs constituted the highest proportion (8.51%) among the three vertical growth patterns. Overall, type 2 spurs were the most common subtype, particularly among hypodivergent individuals, whereas type 3 spurs were the least frequent and relatively more prevalent in the normodivergent group.

No significant association was found between occipital spur type and overjet (p = 0.07) or overbite (p = 0.27). However, a statistically significant difference in spur length was observed among the overbite groups (p = 0.007). Subjects with deep bite exhibited the greatest mean spur length (10.24 ± 2.37 mm), which was significantly greater than that observed in subjects with a normal overbite (8.64 ± 2.19 mm). Post hoc analysis confirmed this finding, demonstrating significantly greater spur length in the deep bite group than in the normal overbite group (p = 0.002; Bonferroni-adjusted p = 0.005).

To assess measurement reliability, all cephalometric measurements were repeated by the examiners after a two-week interval, and reliability was evaluated using the intraclass correlation coefficient (ICC). Both intra-examiner and inter-examiner reliability were excellent, with an ICC of 0.94.

## Discussion

The prevalence of OS has been reported to vary widely in the literature, ranging from 10% to 52%, as documented by Shahar et al. and Gunacar et al. [11,12]. In cadaveric studies, such as the evaluation of 30 skulls, the reported prevalence was at the lower end of this spectrum, approximately 10% [18]. However, this lower incidence is a common finding in cadaveric investigations compared with radiographic studies [10,13]. In contrast, our radiographic assessment of individuals aged 18-24 years demonstrated a considerably higher prevalence of OS (65%). This disparity further supports the premise that radiographic techniques may offer greater sensitivity in detecting OS.

Gender-based analysis revealed a slight male predominance in the occurrence of OS, consistent with previous studies conducted in Turkish and Chinese populations by Gunacar et al., Cheng et al., and Çağlayan [10,11,19]. Despite ethnic differences between our study population and those evaluated previously, the trend of a higher prevalence among males remained consistent. Interestingly, the mean spur length was comparable between males and females in the present study, differing from previous reports that demonstrated significantly longer spurs in males [10-12]. Furthermore, none of the OS in our cohort exceeded 20 mm, consistent with the findings of Gunacar et al. [11,22,23]. These differences may be attributable to racial or anatomical variations among populations.

With respect to occlusion, Çağlayan reported a higher frequency of OS in individuals with crossbites [10]. In the present study, 21 of the 34 individuals with a crossbite exhibited OS. However, no significant association was found between OS and overjet or skeletal malocclusion patterns, suggesting that racial or sample-specific factors may account for these differences. Interestingly, although no significant relationship was observed between occipital spur type and overbite, spur length was significantly greater in subjects with deep bite than in those with normal or open bites. This finding may indicate a mechanical or postural influence on spur development that warrants further investigation. Skeletal malocclusion classification was used in the present study instead of Angle's molar classification because lateral cephalograms do not permit reliable assessment of molar relationships owing to the superimposition of bilateral structures [24]. Although Çağlayan reported similar findings, the analysis in that study was based on molar relationships, making direct comparisons difficult [10].

Regarding morphological variations, type 2 (crest type) was the most frequently observed occipital spur subtype in both males and females, consistent with the findings of Çağlayan [10]. In contrast, Gunacar et al. reported type 1 as the predominant subtype [11]. These discrepancies may reflect population-specific morphological characteristics or differences in classification criteria. A comparison of previous studies evaluating OS is presented in Table 5.

**Table 5:** Compilation of various studies related to occipital spur. CBCT: cone-beam computed tomography; m: male; f: female.

Author	Year	Country	Sample	Spurs	Rate	Frequency	Observed prevalence in males & females	Method of analysis	Mean spur length	Mean spur length (female)	Mean spur (male)	Spur length (<18 years)	Spur length (>18 years)	Spur types			Width	Thickness	Occlusion			Incisor bite					
														Type 1	Type 2	Type 3			Class I	Class II	Class III	Normal	Open bite	Deep bite	Crossbite	Edge to edge	Overjet
Gunacar et al. [11]	2022	Turkey	121	-	14.16	1.3	-	Lateral cephalogram		7.6 ± 3.0	7.4 ± 2.6	7.0 ± 2.5	7.8 ± 2.8	40.8% (n = 49	29.2% (n = 35)	30% (n = 36)	-	-	-	-	-	-	-	-	-	-	-
Cheng et al. [19]	2023	China	451 (f: 196; m: 255)	-	-	-	-	Lateral cephalogram	-	-	-	-	-	-	-	-	-	-	-	-	-	-	-	-	-	-	-
Srivastava et al. [18]	2018	India	30	-	10%	3 out of 30	-	Skull	13.55 ± 1.5	-	-	-	-	-	-	-	19.73 ± 4.85	1.83 ± 0.39	-	-	-	-	-	-	-	-	-
Çağlayan et al. [10]	2024	Turkey	1925 (m: 679; f: 1246)	483	25%	-	m: 37.1%; f: 18.4%	Lateral cephalogram	-	8.23 ± 5.56	11.82 ± 7.11	-	-	133 (27.5%)	247 (51.1%)	103 (21.3%)	f: 11.44 ± 6.84; m: 14.44 ± 7.69	f: 6.64 ± 4.57; m: 9.78 ± 7.22	23.44%	25%	26.95%	27.63%	25.62%	21.93%	29.92%	22.66%	22.17%
Çağlayan et al. [25]	2024	Turkey	526	239	45.69	-	m: 60.28%; f: 35.98%	CBCT	-	-	-	-	-	m: 38 (18.2); f: 42 (13.4%)	m: 38 (18.2%); f: 42 (13.4%)	m: 25 (12%); f: 13 (4.1%)	-	-	-	-	-	-	-	-	-	-	-

Recent studies have highlighted a possible association between OS and postural disorders, particularly those related to prolonged use of handheld electronic devices. Extended screen time, forward head posture, and increased trapezius muscle activity have been proposed as potential contributing factors [16,26]. The greater occipital nerve, which originates from the C2 spinal nerve, courses close to the external occipital protuberance and provides sensory innervation to the posterior scalp. Enlargement of the occipital spur may irritate this nerve, resulting in occipital neuralgia-like symptoms, including radiating pain over the occipital region. In addition, OS have been associated with occipital headaches and referred pain to the shoulder, possibly due to trapezius muscle strain or irritation of the greater occipital nerve [16]. Marshall et al. also reported cases in which surgical excision of painful occipital exostoses resulted in symptomatic relief, supporting a causal role of OS in chronic occipital pain syndromes [17]. In light of these findings, clinicians should consider OS as a potential contributing factor in patients presenting with unexplained occipital or neck pain, particularly after more common causes have been excluded. Accurate diagnosis through radiographic evaluation and careful clinical examination is essential, and management may range from postural correction and physiotherapy to surgical intervention in selected symptomatic cases.

Beyond orthodontics, the morphological characteristics of the external occipital protuberance and occipital spur may have broader applications in medicine, anthropology, and forensic sciences. The external occipital protuberance serves as a stable craniofacial landmark for cephalometric analysis, surgical orientation, and anthropometric measurements. Variations in occipital spur morphology may therefore contribute to craniofacial morphometric assessments and could potentially influence landmark identification in skeletal analyses. Craniofacial anthropometric parameters, including the cephalic index and facial index, are widely employed in population-based studies, ethnic variation research, forensic human identification, and medicolegal investigations to characterize craniofacial diversity and assist in biological profiling [13]. Although the present study did not evaluate these anthropometric relationships, documenting population-specific variations in occipital spur morphology provides baseline anatomical data that may complement future investigations integrating craniofacial indices with occipital morphology. Such multidisciplinary research may improve our understanding of craniofacial variation across different populations and expand the clinical and forensic relevance of occipital spur assessment beyond its orthodontic implications [27,28].

The present study has certain limitations. Its retrospective design, based exclusively on radiographic records, precluded the assessment of behavioral factors, clinical symptoms, and postural habits that may be associated with occipital spur development. Future prospective studies are warranted to investigate these associations further and to explore the clinical implications of OS, particularly among younger individuals and frequent users of digital devices.

## Conclusions

This study demonstrated that the most common occipital spur morphology in the Indian population was type 2 (crest type), with a similar distribution in both males and females. No significant association was observed between occipital spur type and skeletal malocclusion, overjet, or overbite. However, the significantly greater spur length observed in individuals with deep bite warrants further investigation. These findings suggest that occipital spurs may not be directly associated with occlusal patterns, and that postural factors may play a more important role in their development. Given the increasing prevalence of postural disorders in the digital era, further prospective studies are needed to elucidate the etiology, clinical significance, and potential symptomatic associations of occipital spurs.

## Appendices

Sample size calculation

Sample size was determined based on the expected proportion between the two variables, with values estimated from the literature, using the following formula:

$n = \frac{(Z_{1-\alpha/2} + Z_{1-\beta})^2 \left[ p(1-p) + q(1-q) \right]}{(p-q)^2}$

$n = \frac{(1.96 + 0.84)^2 [0.35]}{(0.22)^2}$

Where *Z1−α/2* is the z-value corresponding to the significance level (alpha error), a constant equal to 1.96. *Z1−β* is the z-value corresponding to the power of the study (beta error), a constant equal to 0.84. *p* and *q* are the proportions of the variable, with values obtained from the parent article (Chow et al. [29]).

Assumptions used in the calculation:

Power of the study: 80%

Type I error (α): 5%

Type II error (β): 20%

Degree of association between the variables: 0.22

Based on this calculation, a minimum of 60 subjects per group would provide adequate external validity for the present study.
